# Towards sustainable development in China: How do green technology innovation and resource misallocation affect carbon emission performance?

**DOI:** 10.3389/fpsyg.2022.929125

**Published:** 2022-07-19

**Authors:** Mingyue Du, Qingjie Zhou, Yunlai Zhang, Feifei Li

**Affiliations:** ^1^School of Economics, Beijing Technology and Business University, Beijing, China; ^2^Institute of New Commercial Economy, Beijing Technology and Business University, Beijing, China; ^3^School of International Economics and Management, Beijing Technology and Business University, Beijing, China

**Keywords:** green technology innovation, carbon emission performance, China, capital misallocation, labour misallocation

## Abstract

Green technology innovation is an effective way through which to achieve carbon neutrality and sustainable development. Based on provincial panel data of 30 provinces in China from 2005 to 2018, this work examines the tripartite relationship among green technology innovation, resource misallocation, and carbon emission performance by constructing panel regression models and a dynamic threshold panel model. The research results show that green technology innovation significantly improves carbon emission performance. Further analysis shows that both capital and labour misallocation have a negative impact on carbon emission performance and hinder the contribution of green technology innovation to the improvement of carbon emission performance. The regression results show that there is a threshold effect of green technology innovation on carbon emission performance: as the degree of resource misallocation increases, the positive impact of green technology innovation on carbon emission performance gradually decreases. This study provides an important reference for policy-makers in implementing policies to improve carbon emission performance. Policy-makers should continue to promote the level of green technology innovation and improve the efficiency of labour and capital allocation.

## Introduction

Climate change has become the most serious challenge to global sustainable development (Nordhaus, 2019; Heyd, 2021; Zhou et al., 2022). The massive energy consumption brought about by accelerated urbanization and industrialization has led to increased carbon emissions with serious consequences for living systems and the global climate as a whole (Wu et al., 2020; Sufyanullah et al., 2022). The United Nations Framework Convention on Climate Change (UNFCCC), Kyoto Protocol, and Paris Agreement have established a framework for global climate governance and low-carbon green development, among which the Paris Agreement clarifies the common but differentiated responsibilities of all countries. Statistics from the Statistical Review of World Energy show that China’s CO2 emissions in 2020 were 9,899.3 million tons, accounting for 30.7% of the world total. As the largest carbon emitter in the world, China faces enormous challenges in many areas, including climate change, economic transformation, environmental protection, and green development (Liu et al., 2022). China has assumed the responsibility of being a major country in terms of addressing climate change and has pledged to achieve a carbon emission peak by 2030 and reduce carbon emission intensity by 60–65% compared with the 2005 levels. To achieve this goal, China has taken many energy conservation and emission reduction measures, such as optimizing and adjusting the energy structure, improving energy utilization efficiency, promoting the economic and intensive use of natural resources, and launching online trading in the national carbon market (Yang et al., 2021b). There is no doubt that the effective implementation of energy conservation and emission reduction will inevitably lead to carbon peaking and carbon neutrality (Sun et al., 2022; Zeng et al., 2022).

Green technology innovation and rational resource allocation are the key approaches to solving carbon emissions and environmental problems (Razzaq et al., 2021). Green technology is a modern technology system that is in coordination with the ecological environment system, including pollution prevention technology, source emission reduction technology, waste reduction technology, recycling production technology, green products, and purification technology (Zhang and Li, 2022). Moreover, green technology has good positive externalities and can produce good social and environmental benefits. Green technology innovation can improve the utilization efficiency of raw materials and energy, reduce the cost of resource utilization, and alleviate environmental pollution, which are all conducive to the realization of green, low-carbon, and sustainable development (Lv et al., 2021; Chen et al., 2022). Moreover, rational resource allocation can reduce resource waste and improve resource utilization efficiency. However, due to the impact of the planned economy, China is currently facing a serious overcapacity problem. Resource misallocation can be found in different ownership structures, regions, and industries, in turn reducing total factor productivity and increasing carbon emissions and environmental pollution. In addition, China has a vast territory, and its resource endowment varies greatly from place to place. Local protectionism, market segmentation, and local market access systems severely restrict the free flow of resources and hinder the smooth economic cycle (Hao et al., 2020a).

According to neoclassical economic growth theory, economic growth depends on the advancement of technological factors and the increase in the input of labour and capital factors (Solow, 1957). Due to global warming and green development, China has insisted on promoting green and low-carbon transformation and development. Therefore, in such a limited energy environment, carbon emission and energy factors are included in the TFP growth calculation, and the total factor carbon emission performance obtained can accurately estimate the economic development mode (Xu et al., 2021). Referring to the measurement mode of carbon emission performance (Xu et al., 2021), this paper focuses on the impact of green technology innovation and resource misallocation on carbon emission performance.

The literature on green technology innovation, resource misallocation, and carbon emissions is relatively abundant; however, previous studies lacked a systematic incorporation of green technology innovation, resource misallocation, and carbon emission performance into the same analytical framework. Does green technology innovation help improve carbon emission performance? What effect does resource misallocation have on carbon emission performance? Is there a threshold effect on the impact of green technology innovation on carbon emission performance?

To answer the above questions, this work systematically studies green technology innovation, resource misallocation, and carbon emission performance using Chinese provincial panel data from 2005 to 2018. Based on existing research, this paper includes the following three main innovations. First, we put green technology innovation, resource misallocation, and carbon emission performance into the same analytical framework to better study their tripartite relationship. Second, through a moderating effect model, we separately examine the moderating effects of capital and labour misallocation on the impact of green technology innovation on carbon emission performance. Third, a dynamic threshold model is used to test the impact of green technology innovation on carbon emissions through the use of capital misallocation and labour misallocation threshold variables.

The remainder of the article is organized as follows. Section “Literature review” reviews the relevant literature. Section “Methodology and data” presents the model and data. Section “Empirical results and discussion” presents mainly the empirical results and discussion. The conclusions are presented in the final section.

## Literature review

### Green technology innovation and environmental pollution

With the development of science and technology, green technology has gone through various: terminal treatment technology, waste-free technology, clean technology, renewable energy technology, and pollution prevention technology (Fujii and Managi, 2019). At present, green technology can be regarded as a type of technology that improves the environment, provides ecological protection, and promotes sustainable development in the fields of energy conservation, environmental protection, clean energy, cleaner production, and the circular economy (Wang et al., 2019; Sinha et al., 2020). Climate change and environmental issues have become increasingly prominent, and the relationship between green technology innovation and the environment has received widespread attention. However, there is no consensus on the environmental impact of technological innovation (Ren et al., 2022a), as previous studies have very different views of this impact. Some scholars believe that green technology innovation can reduce carbon emissions and environmental pollution. For example, Álvarez-Herránz et al. (2017) studied 28 OECD countries and found that energy innovation helps reduce greenhouse gas emissions. After researching select OECD economies, Ganda (2019) argued that improving renewable energy technology and spending on R&D could reduce carbon emissions, suggesting that countries should promote the use of green energy and prioritize R&D activities. Moreover, Yuan et al. (2022) argued that green innovation could reduce carbon dioxide emissions and that when institutional quality is higher, green innovation has a stronger reduction effect on carbon dioxide emissions. After constructing a spatial Durbin model and a threshold model, Ren et al. (2022a) suggested that the development of internet technology reduces not only local environmental pollution but also that in adjacent areas.

However, other viewpoints hold that the effects of green technology innovation on carbon emissions and the environment are not significant. Through a study of 71 economies, Du et al. (2019) found that for those with lower income levels, green technology innovation makes no significant contribution to reducing carbon emissions. Wang and Zhu (2020) suggested that the effect of energy technology innovation on carbon dioxide emission reduction be differentiated. Renewable energy technology innovation contributes to carbon dioxide emission reduction, but fossil energy technology innovation does not play a role in reducing carbon emissions. Furthermore, Adebayo and Kirikkaleli (2021) found that technological innovation increased Japan’s CO2 emissions in both the short and medium terms.

### Research on resource misallocation

In an economy where resources can flow freely enough to achieve Pareto optimality, efficient resource allocation is said to exist, and resource misallocation denotes a deviation from this ideal state (Berthou et al., 2019; Wang et al., 2021). Resource misallocation usually includes capital misallocation, labour misallocation, land misallocation, and other types of misallocation (Hao et al., 2020a). Specifically, capital misallocation occurs mainly because funds, as scarce resources, cannot flow freely to industries and regions that can generate higher economic benefits, which violates the objective laws of economics (Huang et al., 2021). The main reasons for capital misallocation are financial frictions, corporate investor preferences, government subsidies, credit bias among banks, financial repression, and insufficient corporate financial liquidity and financial pledge capacity.

Labour is an important production factor, and the labour market is a market involving two-way choices. Workers choose high-quality enterprises based on their own experience, education, expertise, preferences, and career ideals. Companies usually select excellent employees based on their differentiated characteristics. Therefore, due to information asymmetry, differences in the capabilities of firms and workers can lead to labour misallocation (Kong et al., 2021). Moreover, there is a labour shortage in economically developed areas along the eastern coast of China, while in the inland areas of the western region that have large populations, there is a labour surplus. The optimal allocation of labour resources is from west to east. However, factors such as the household registration system hindering free labour movement eventually lead to labour misallocation.

In summary, capital misallocation and labour misallocation have caused many problems, such as aggravating the imbalance of regional and industrial development, reducing total factor productivity, bringing about excess capacity, economic losses, and environmental pollution, hindering the smooth economic cycle, and restricting economic development (Wang et al., 2020; Hao et al., 2020a).

### Research on carbon emissions

As a key factor in climate change, carbon emissions have attracted extensive attention. At present, studies on carbon emissions focus mainly on the measurement method, influencing factors, and efficiency of carbon emissions, as well as the trading of carbon emission rights (Dauda et al., 2021; Wu et al., 2021). Moreover, from the perspective of carbon emission measurement, there are four main methods: the actual measurement method, mass balance method, factor decomposition method, and IPCC inventory method (Krisnawati et al., 2021; Nie et al., 2022). Specifically, the actual measurement method is used mainly in natural ecosystems, with total emissions being calculated according to the flow, speed, concentration, etc., of the emission gas collected through observation. The mass balance method is used mainly in industrial production processes to calculate gas emissions according to the law of the conservation of the input and output of substances. Both the factor decomposition method and IPCC inventory method are used for fossil energy consumption, with the former being used to quantitatively estimate and analyse carbon emissions based on the construction of mathematical models and the latter being based mainly on energy performance consumption. In actual research, most scholars have used the “2006 IPCC Guidelines for National Greenhouse Gas Inventories,” published by the IPCC, to measure carbon emissions (Wu et al., 2020; Hao et al., 2021). Moreover, regarding the influencing factors of carbon emissions, previous studies have used mainly three methods—index decomposition analysis (IDA), structural decomposition analysis (SDA), and stochastic impacts by regression on population, affluence, and technology (STIRPAT)—to analyse the influencing factors of carbon emissions. Among them, the decomposition method can determine the actual contribution of each factor to carbon emissions. Additionally, although the STIRPAT model can flexibly incorporate a variety of influencing factors into the model for analysis according to corresponding theory, it can estimate only the elastic coefficient of each influencing factor. In addition, existing studies have attributed the factors affecting carbon emissions mainly to economic development, the energy structure, energy efficiency, population size, the industrial structure, urbanization level, and technological progress.

In general, scholars have carried out a series of studies on green technology innovation, carbon emissions, resource misallocation, and environmental pollution (Hao et al., 2020a; Du et al., 2021; Ren et al., 2021; Chen et al., 2022). However, a more in-depth and systematic analysis of the tripartite relationship among green innovation, resource misallocation, and carbon emission performance is lacking. First, few studies have explored the impact of green technology innovation on carbon emission performance from the resource allocation perspective. Second, we examine whether capital and labour misallocation weaken the impact of green technology innovation on carbon emission performance. In addition, we examine whether there is a threshold effect between the impact of green technology innovation and carbon emission performance. If such an effect exists, then an effective adjustment mechanism needs to be established in the process of developing green technology innovation. Currently, with the acceleration of urbanization, resource misallocation, and environmental pollution are becoming increasingly serious (Hao et al., 2020a; Yang et al., 2021a). Therefore, this article analyses green technology innovation, resource misallocation, and carbon emission performance from the above perspectives.

## Methodology and data

### Econometric methodology

#### Basic linear model

This paper focuses on the tripartite relationship among green technology innovation, resource misallocation, and carbon emission performance. Therefore, an econometric model is constructed as shown in Equation (1). Considering that we focus on capital misallocation and labour misallocation, this work constructs the following econometric models, as shown in Equations (2–4):


(1) $$\begin{array}{c} cep_{it}=\beta_{0}+\beta_{1}lngti_{it}+\beta_{2}pgdp_{it}+\beta_{3}ce_{it}+\\ \beta_{4}open_{it}+\beta_{5}gov_{it}+\beta_{6}fdi_{it}+\alpha_{i}+v_{t}+\varepsilon_{it} \end{array}$$

(2) $$\begin{array}{c} cep_{it}=\beta_{0}+\beta_{1}rk_{it}+\beta_{2}pgdp_{it}+\beta_{3}ce_{it}+\beta_{4}open_{it}+\\ \beta_{5}gov_{it}+\beta_{6}fdi_{it}+\alpha_{i}+v_{t}+\varepsilon_{it} \end{array}$$

(3) $$\begin{array}{c} cep_{it}=\beta_{0}+\beta_{1}rl_{it}+\beta_{2}pgdp_{it}+\beta_{3}ce_{it}+\beta_{4}open_{it}+\\ \beta_{5}gov_{it}+\beta_{6}fdi_{it}+\alpha_{i}+v_{t}+\varepsilon_{it} \end{array}$$

(4) $$\begin{array}{c} cep_{it}=\beta_{0}+\beta_{1}rk_{it}+\beta_{2}rl_{it}+\beta_{3}pgdp_{it}+\beta_{4}ce_{it}+\\ \beta_{5}open_{it}+\beta_{6}gov_{it}+\beta_{7}\ln fdi_{it}+\alpha_{i}+v_{t}+\varepsilon_{it} \end{array}$$

where $cep_{it}$ represents carbon emission performance; $gti_{it}$ denotes green technology innovation; $rk_{it}\;and\;rl_{it}$ represent capital misallocation and labour misallocation, respectively; *i* and *t* denote province and time, respectively; and $\varepsilon_{it}$ is the random disturbance term. The control variables include economic development ($pgdp_{it}$), carbon emissions ($ce_{it}$), trade openness ($open_{it}$), government intervention ($gov_{it}$), and foreign direct investment ($fdi_{it}$).

#### Dynamic threshold panel model

To analyse the threshold effect between green technology innovation and carbon emission performance under different degrees of capital misallocation and labour misallocation, we refer to the research of Ren et al. (2022b) and use a dynamic threshold model to separately discuss their impact on GTFEE with corruption and market segmentation as threshold variables. The threshold regression model is established as follows:


(5) $$\begin{array}{c} cep_{it}=\beta_{0}+\beta_{1}lngti_{it}\cdot I\left(q_{it}\leq c\right)+\\ \beta_{2}lngti_{it}\cdot I\left(q_{it}>c\right)+\overset{5}{\underset{k=1}{\sum }}\beta_{k}X_{kit}+\alpha_{i}+v_{t}+\varepsilon_{it} \end{array}$$

where $q_{it}\;$represents the threshold variable, which, here, refers to$\;rk_{it}\;and\;rl_{it}$, I(∙) represents the indicator function, and c is the specific threshold value.

### Data

#### Carbon emission performance

Referring to Xu et al. (2021), combined with the nonangular and nonradial DDF proposed by Zhou et al. (2012), a DEA calculation model of the environmental total factor productivity growth index based on overall technology is constructed, and then, the carbon emission performance of 30 provinces in China from 2005 to 2018 is measured.

#### Green technology innovation

Green technology innovation can apply green technology to all stages of production, giving play to the spillover effects of innovation and the environment (Du et al., 2021). This paper uses the total number of green patent grants in each province to measure green technology innovation. Specifically, according to the International Patent Classification Code of Green Patents provided in the Green Patents Inventory issued by the World Intellectual Property Organization, we screen green patents from the patent database of the State Intellectual Property Office of China based on the date of patent authorization to measure green technology innovation in each province and process them logarithmically (Du et al., 2019).

**Figure 1:**
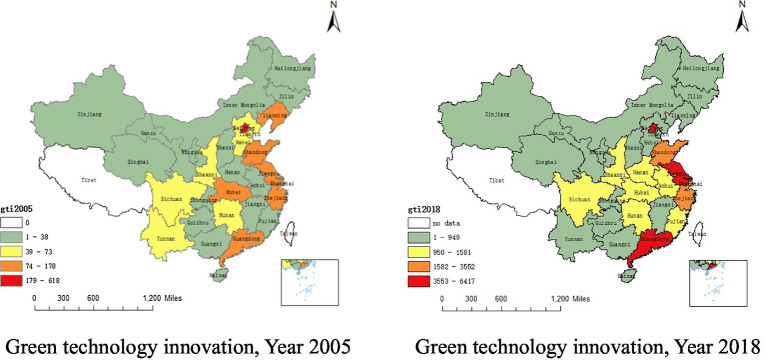
China’s green technology innovation in the year 2005 and 2018.

Figure 1 shows China’s green technology level in 2005 and 2018. Overall, Beijing, Guangzhou, Jiangsu and Shanghai are at the forefront of the country’s green technology levels. Unlike in Xinjiang, Qinghai, northeast regions, and other regions where the level of green technology innovation has not changed much, in Hebei, Hubei, Liaoning and Yunnan, the level has declined, and in Guangzhou, Jiangsu, Anhui and Fujian, the level has showed an upwards trend.

#### Resource misallocation

Resource misallocation not only reduces total factor productivity but also affects carbon emission performance. Referring to the theoretical framework of scholars such as Hsieh and Klenow (2009) and Hao et al. (2020a), this work assumes that a factor input has a distorted competitive market and defines the absolute distortion coefficient of the capital factor (*kr*) and labour factor (*lr*) as in Equation (6) as follows:


(6) $$\gamma_{K}=\frac{1}{1+\tau_{Ki}},\gamma_{L}=\frac{1}{1+\tau_{Li}}$$

where $\gamma_{k}$ and $\gamma_{L}$ represent the addition of factor inputs. When there are no capital misallocation and labour misallocation, $\tau_{Ki}=0$, and $\tau_{Li}=0$, and then, $\gamma_{k}=1$, and $\gamma_{L}=1$, which means that there is no capital factor and labour factor input addition distortion.

This work further assumes that each province has a C-D production function with constant returns to scale, as shown in Equation (7), in which the real GDP in 2005 is used as the base period to represent the output variable *Y_it_*. The capital and labour factor inputs are represented by *K_it_* and *L_it_*, respectively, where the former is represented by the fixed capital stock of each province, and the latter is represented by the average annual employment of each province. In addition, on this basis, Equation (7) is calculated according to the Solow residual method, the logarithm of both sides of the equation is taken, individual and time effects are controlled in the model, and then, Equation (8) is obtained. Moreover, we add the interaction term of individual dummy variables and explanatory variables to measure the input–output elasticities *β_Ki_* of the capital factor and *β_Li_* of the labour factor in region i. On this basis, the indicators are substituted into Equation (9) to calculate the relative distortion coefficients ${\overset{⌢}{\gamma }}_{K}$ and ${\overset{⌢}{\gamma }}_{L}$ to approximately replace $\gamma_{k}$ and $\gamma_{L}$, respectively. Finally, the index data are substituted into Equation (6) to obtain the capital and labour misallocation indices $\tau_{Kit}$ and $\tau_{Lit},$ respectively.


(7) $$Y_{it}=AK_{it}^{\beta_{Ki}}L_{it}^{\beta_{Li}}$$

(8) $$\ln\left(Y_{it}/L_{it}\right)=\ln A+\beta_{Ki}\ln\left(K_{it}/L_{it}\right)+\mu_{i}+\lambda_{t}+\varepsilon_{it}$$

(9) $${\overset{⌢}{\gamma }}_{K}=\left(\frac{K_{i}}{K}\right)/\left(\frac{s_{i}\beta_{Ki}}{\beta_{K}}\right),{\overset{⌢}{\gamma }}_{L}=\left(\frac{L_{i}}{L}\right)/\left(\frac{s_{i}\beta_{Li}}{\beta_{L}}\right)$$

where *K_i_* represents the capital stock of region *i*, *K* represents the total capital stock of the whole country, and $\frac{K_{i}}{K}$ represents the ratio of the capital actually used in region *i* to the national capital stock. $s_{i}\frac{\gamma_{i}}{\gamma }$ represents the output value of region *i*. *Y_i_* accounts for the share of the total national output value *Y*. Taking the capital factor as an example, $\beta_{K}=\overset{N}{\underset{i}{\sum }}s_{i}\beta_{Ki}$ represents the total input–output elasticity of capital in each region after weighted summation, and $\frac{s_{i}\beta_{Ki}}{\beta_{K}}$ measures the theoretical proportion of capital that region *^i^* should use under the ideal situation of efficient capital allocation. From the calculation formula of the relative distortion coefficient, it can be seen that if ${\overset{⌢}{\gamma }}_{K}>1$, then the actual capital used in area *^i^*D exceeds the theoretical value, and the capital is overallocated; otherwise, the actual capital used in area *i* is insufficient.

**Figure 2:**
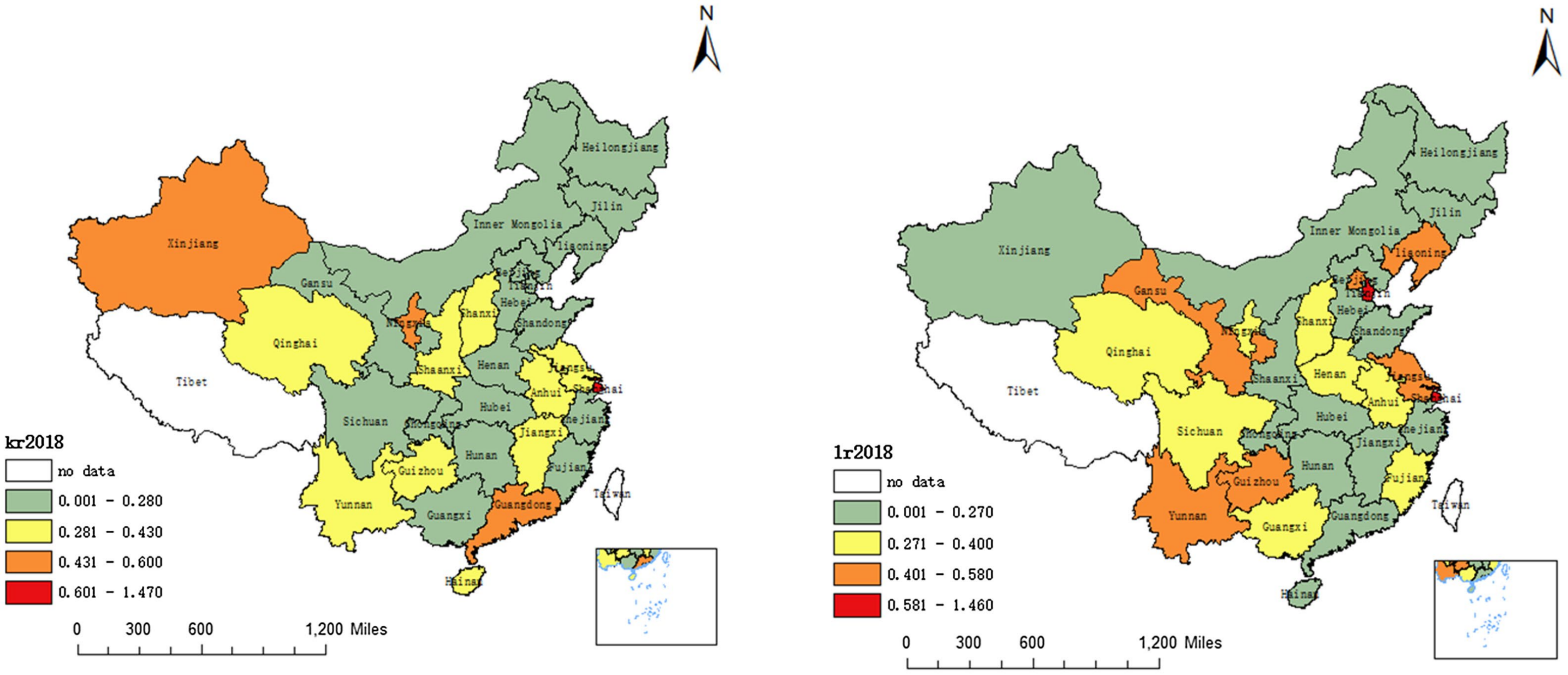
China’s capital misallocation and labour misallocation in the year 2018.

Figure 2 shows China’s capital misallocation and labour misallocation in 2018. In Xinjiang, Ningxia, Shanghai and Guangdong, capital misallocation is quite serious, followed by Qinghai, Yunnan, Shanxi, Shaanxi, Guizhou, Jiangxi and Anhui, while that in other regions is relatively mild. In 2018, the degree of labour misallocation in Shaanxi, Inner Mongolia, Xinjiang, Heilongjiang, Jilin, and Guangdong is relatively low, while that in Shanxi, Qinghai, Ningxia, Henan, Anhui, Fujian, Sichuan, Guangxi, and Qinghai is more serious. Furthermore, Beijing, Tianjin, Liaoning, Jiangsu, Shanghai, Gansu, Guizhou and Yunnan have the highest degree of labour misallocation.

#### Control variables

##### Level of economic development (pgdp)

Rapid economic development requires many resources, thus impacting the environment and carbon emission performance. This study uses *per capita* GDP to represent the economic development level of each province (Wan and Sheng, 2022).

##### Environmental regulation (eru)

The government’s governance of the environment regulates the technological innovation, resource allocation, and carbon emission behaviour of enterprises, thereby affecting their carbon emission performance. This study uses the ratio of completed investment in industrial pollution control to GDP to represent the level of environmental regulation (Hao et al., 2020b).

##### Level of openness (open)

The rapid development of foreign trade has effectively promoted economic development. However, foreign trade involves a large amount of carbon emissions, which have a serious environmental impact. Therefore, this study uses the proportion of total import and export trade to GDP to measure the level of openness (Ren et al., 2021).

##### Government intervention (gov)

Government intervention in economic activities through fiscal and monetary policies can affect green technology innovation, resource allocation, and carbon emission performance. Therefore, this study uses the proportion of the general budget expenditure of local finance to GDP to represent the level of government intervention (Su et al., 2021).

##### Foreign direct investment (fdi)

For regions with high levels of technological innovation and pollution, emission rights and innovation are more inclusive and thus more likely to attract foreign direct investment, further affecting carbon performance; therefore, this study uses the actual utilization of foreign direct investment to measure the level of foreign direct investment (Liu et al., 2018).

#### Data sources

The sample in this paper includes the panel data of 30 provinces in Mainland China (excluding Tibet) from 2005 to 2018. The data sources are the *China Statistical Yearbook, China Environmental Statistical Yearbook, China Energy Statistical Yearbook, China Energy Database, Easy Professional Superior,* and *Wind Database*. This paper addresses the issue of missing data with the imputation method, finally obtaining 420 observations. The descriptive statistics of each variable are shown in Table 1.

**Table 1:** The statistical description of variables.

Variable	Obs	Mean	*SD*	Min	Max	Unit
cep	420	−0.052	2.201	−10.02	8.730	-
gti	420	5.192	1.664	0.000	8.831	-
kr	420	0.241	0.181	0.000	1.470	-
lr	420	0.417	0.408	0.000	3.050	-
pgdp	420	3.849	2.502	0.522	15.31	10,000 yuan
eru	420	0.002	0.001	0.000	0.011	%
open	420	0.320	0.374	0.018	1.711	%
gov	420	0.235	0.108	0.092	0.758	%
fdi	420	4.104	4.684	0.001	22.57	10 billion yuan

## Empirical results and discussion

### Discussion of the effect of green technology innovation on carbon emission performance

The estimated results of the effect of green technology innovation on carbon emission performance are reported in Table 2. The regression results of OLS, FE, and RE regressions show that the coefficients of green technology innovation are highly positive, indicating that it can effectively improve carbon emission performance. Considering the endogeneity and heteroskedasticity problems in the model, generalized method of moment’s estimation is selected to solve this problem. The regression results show that the first-order lag of L.cep is significantly positive, suggesting that carbon emission performance has notable path dependence. Furthermore, no significant change in the sign and significance level of the regression coefficient is found in the SYS-GMM estimation results, and thus, the above conclusion is still valid, mainly because green innovation greatly stimulates green demand, which in turn promotes the development of green industries (Zhu and Tan, 2022). Moreover, the improvement of green technology can reduce resource energy consumption and production costs, leading to the spontaneous flow of production factors to high-productivity sectors, improving industrial production efficiency and resource utilization efficiency, reducing pollution emissions, and enhancing carbon emission performance (Yuan et al., 2022).

**Table 2:** The regression results of green technology innovation on carbon emission performance.

Variables	OLS	FE	RE	SYS-GMM
*L.cep*				0.333^***^
				(6.947)
*lngti*	0.320^***^	0.454^*^	0.311^**^	0.361^**^
	(2.880)	(1.911)	(2.386)	(1.986)
*pgdp*	0.156^**^	0.425^***^	0.169^**^	0.129
	(2.246)	(2.809)	(2.108)	(0.994)
*eru*	−3.039^***^	−3.132^***^	−2.956^***^	−3.220^***^
	(−4.170)	(−3.380)	(−3.630)	(−3.945)
*open*	−0.610^*^	3.573^***^	−0.493	−1.167
	(−1.847)	(3.425)	(−1.244)	(−1.144)
*gov*	−1.356	−11.493^***^	−2.005	−3.074^**^
	(−1.236)	(−4.312)	(−1.521)	(−2.248)
*fdi*	−0.026	0.062	−0.018	−0.041
	(−0.944)	(1.245)	(−0.525)	(−0.808)
*_cons*	−1.189^**^	−2.227^***^	−1.120^*^	−0.501
	(−2.002)	(−3.073)	(−1.729)	(−0.528)
*AR(1)*				−3.46
				[0.001]
*AR(2)*				1.21
				[0.225]
*Hansen test*				23.58
				[0.958]
*R-squared/Wald test*	0.215	0.118	0.213	351.79^***^
*N*	420	420	420	420

*** *p* < 0.01;

** *p* < 0.05;

* *p* < 0.1.

Figures in () are the *t* values of the coefficients, and figures in [] are the *p* values of the corresponding test statistics. L. is the lag of the variable.

### Discussion of the effect of resource misallocation on carbon emission performance

As mentioned above, this study analyses mainly capital misallocation and labour misallocation. The samples selected in this paper have the data structure characteristics of “large N and short T.” Considering endogeneity and heteroskedasticity problems, system generalized method of moments estimation is used to analyse the impact of resource misallocation on carbon emission performance. To ensure the validity of the analysis results, we examine the effects of capital misallocation and labour misallocation on carbon emission performance. The regression results in Table 3 show that by gradually increasing the values of the control variables, the regression coefficients of capital misallocation and labour misallocation both become significantly negative, indicating that neither capital misallocation nor labour misallocation is conducive to promoting carbon emission performance. The main reason for this is perhaps that the inverse efficiency flow of factor resources hinders the flow of resources to high-efficiency sectors and enterprises due to resource misallocation (Bian et al., 2019), resulting in reduced resource allocation efficiency, production efficiency, and carbon emission performance, consistent with the findings of Yang et al. (2022). However, the factors that cause resource misallocation include enterprise ownership type, enterprise scale, market segmentation, local protection, and government intervention. Studies have shown that these factors are not conducive to improving resource allocation efficiency, achieving energy conservation and emission reduction, and improving environmental and carbon emission performance (Zhou et al., 2022).

**Table 3:** The regression results of resource misallocation on carbon emission performance.

Variables	SYS-GMM	SYS-GMM	SYS-GMM	SYS-GMM	SYS-GMM	SYS-GMM
*L.cep*	0.186^***^	0.487^***^	0.403^***^	0.015^**^	0.460^***^	0.363^***^
	(5.605)	(11.114)	(8.573)	(2.264)	(11.098)	(8.121)
*kr*	−2.211^***^	−0.732^**^	−8.043^**^			
	(−3.421)	(−2.216)	(−2.394)			
*lr*				−2.601^***^	−1.079^***^	−2.612^**^
				(−3.986)	(−2.950)	(−2.056)
*pgdp*		0.146^***^	0.475^***^		0.238^***^	0.326^***^
		(5.143)	(3.451)		(6.993)	(3.974)
*eru*		−1.499^***^	−2.669^***^		−3.053^***^	−4.036^***^
		(−4.684)	(−2.770)		(−6.740)	(−6.950)
*open*			0.212			1.929
			(0.206)			(0.676)
*gov*			−0.832			0.951
			(−0.495)			(0.269)
*fdi*			−0.118^**^			−0.082
			(−2.537)			(−1.230)
*_cons*	0.655^***^	−0.004	1.289	1.037^***^	0.160	0.044
	(3.925)	(−0.027)	(1.364)	(4.922)	(0.614)	(0.039)
*AR(1)*	−3.25	−3.61	−3.08	−2.91	−3.49	−3.27
	[0.001]	[0.000]	[0.002]	[0.004]	[0.000]	[0.001]
*AR(2)*	0.81	1.30	1.31	0.29	1.37	1.35
	[0.418]	[0.194]	[0.189]	[0.772]	[0.169]	[0.176]
*Hansen test*	29.60	26.60	21.09	28.01	26.16	26.18
	[0.978]	[0.982]	[0.996]	0.793	0.942	0.908
*Wald test*	684.97^***^	523.14^***^	1000.50^***^	47.56^***^	2536.38^***^	387.73^***^
*N*	420	420	420	420	420	420

*** *p* < 0.01;

** *p* < 0.05.

Figures in () are the *t* values of the coefficients, and figures in [] are the *p* values of the corresponding test statistics. L. is the lag of the variable.

### Discussion of the moderating effect of resource misallocation

Capital and labour resources play an important role in green technology breakthroughs and green product marketization. If elemental resources are misallocated, and then green technology innovation faces difficulty in promoting carbon emission performance. To explore the effect of the role of resource misallocation in the impact of green technology innovation on carbon emission performance, this study uses three moderating variables to test the moderating role of resource misallocation in this relationship. These variables are the interaction term of green technology innovation and capital misallocation, that of green technology innovation and labour misallocation, and that of green technology innovation, capital misallocation, and labour misallocation. The regression results in Table 4 show that the coefficients of the three interaction terms are all significantly negative, at −3.363, −4.434, and −0.519. The main reason for this is that green technology innovation requires higher efficiency in the creation and use of green knowledge and more capital and talent. If resources are misallocated, then the allocation efficiency of green innovation resources is reduced and carbon emission performance is thus affected. Moreover, the restrictions on labour mobility imposed by the household registration system also limit employment options for green innovative talent. Labour misallocation leads to the inability of green innovation R&D talent to receive reasonable remuneration, thereby weakening the motivation to carry out green innovation activities. Furthermore, if capital misallocation exists, then government intervention in the green credit decisions of financial institutions guides the flow of green credit to low-risk, high-yield constructive projects, making it easier for high-risk, low-return green innovation projects to be faced with financing difficulties. Due to the high demand for green innovation funds and the tightening constraints of green credit funds, enterprises often choose to intensively use tangible elements, such as capital and labour, to carry out production activities, which affects their motivation to carry out green innovation activities. Such a situation is detrimental to the efficient allocation of green innovation resources and adversely affects carbon emission performance.

**Table 4:** The regression results of moderating effect.

Variables	SYS-GMM	SYS-GMM	SYS-GMM
*L.cep*	0.350^***^	0.258^**^	0.444^***^
	(5.881)	(2.390)	(7.209)
*lngtikr*	−3.237^**^		
	(−2.268)		
*lngtilr*		−4.434^**^	
		(−2.312)	
*lngtikrlr*			−0.455^***^
			(−2.730)
*lngti*	0.984	1.756^**^	0.381
	(1.594)	(2.281)	(1.267)
*kr*	17.771^*^		4.048
	(1.870)		(1.477)
*lr*		29.647^**^	−3.356^**^
		(2.238)	(−2.005)
*pgdp*	0.372	0.377	0.209
	(1.130)	(1.528)	(1.469)
*eru*	−2.063^***^	−1.362	−1.620^**^
	(−4.581)	(−0.883)	(−2.573)
*open*	0.509	−1.206	1.709
	(0.592)	(−0.275)	(1.389)
*gov*	9.438	1.326	−5.384^*^
	(1.263)	(0.345)	(−1.767)
*fdi*	0.007	−0.043	−0.161^***^
	(0.098)	(−0.346)	(−2.746)
*_cons*	−8.842^**^	−12.241^**^	−0.303
	(−2.439)	(−2.354)	(−0.208)
*AR(1)*	−2.90	−2.97	−3.42
	[0.004]	[0.003]	[0.001]
*AR(2)*	1.35	1.16	1.23
	[0.176]	[0.246]	[0.219]
*Hansen test*	20.26	21.08	18.01
	[0.999]	[0.999]	[0.875]
*Wald test*	1275.00^***^	200.32^***^	1144.95^***^
*N*	420	420	420

*** *p* < 0.01;

** *p* < 0.05;

* *p* < 0.1.

Figures in () are the *t* values of the coefficients, and figures in [] are the *p* values of the corresponding test statistics. L. is the lag of the variable.

### Discussion of the regression results of the dynamic threshold panel model

The previous empirical results show that resource misallocation has a negative moderating effect on the relationship between the promotion of green technology innovation and carbon emission performance. Is there any heterogeneity in the impact of green technology innovation on carbon emission performance under different resource misallocation levels? To answer this question, a dynamic GMM threshold panel model is adopted to examine the nonlinear effects of green technology innovation on carbon emission performance at different levels of capital misallocation and labour misallocation. The GMM method is used to construct moment estimation conditions, and the threshold model is embedded in the GMM model. The threshold value is determined by the grid search algorithm, thereby obtaining a dynamic GMM panel (Hao et al., 2020a). The test results are shown in Table 5.

**Table 5:** The threshold tests.

Variable	Threshold value	Wald	*p* value	95% CI	
Capital misallocation	0.330	0.805	0.000	0.040	0.550
Labour misallocation	0.080	1.523	0.000	0.050	1.220

Table 5 shows the results of the threshold value and confidence intervals for capital misallocation (*k_r_*) and labour misallocation (*l_r_*). According to the Wald statistics and their *p* values, all the dynamic threshold models with different threshold variables exhibit significant threshold effects at the 1% significance level. Therefore, threshold effects exist. Furthermore, the impact of green technology innovation on carbon emission performance exhibits a nonlinear feature due to resource misallocation.

Table 6 shows the correlation test results of the two-step GMM threshold model regression. According to the results of the correlation test of the residual sequence, there is no second-order autocorrelation for the random error term. The Hansen test results show that the selection of instrumental variables is effective. Therefore, the dynamic threshold regression results in this study are found to be credible.

**Table 6:** The results of threshold model.

Variable	Kr	lr
	SYS-GMM	SYS-GMM
*L.cep*	0.287^***^	0.287^***^
	(7.46)	(7.39)
*pgdp*	−0.567^***^	−0.597^***^
	(−3.37)	(−4.44)
*eru*	−5.794^***^	−5.677^***^
	(−6.23)	(−6.11)
*open*	1.411^***^	1.375^***^
	(4.29)	(3.81)
*gov*	−3.436^*^	−3.883^***^
	(−1.75)	(−2.94)
*fdi*	−0.217^***^	−0.217^***^
	(−4.97)	(−5.04)
$kr_{it}\leq C$	0.975^***^	
	(3.64)	
$kr_{it}>C$	0.947^***^	
	(2.97)	
$lr_{it}\leq C$		1.038^***^
		(3.36)
$lr_{it}>C$		1.012^***^
		(4.05)
*_cons*	−0.658	−0.696
	(−0.68)	(−0.76)
*AR (1)*	−2.91	−2.95
	[0.004]	[0.003]
*AR (2)*	0.74	0.71
	[0.461]	[0.475]
*Hansen test*	20.82	20.40
	[0.470]	[0.496]
*Wald test*	1168.26^***^	1622.20^***^
	[0.000]	[0.000]
*N*	420	420

*** *p* < 0.01;

* *p* < 0.1.

Figures in () are the *z* values of the coefficients, and figures in [] are the *p* values of the corresponding test statistics. L. is the lag of the variable.

First, we use capital misallocation as a threshold variable to investigate the impact of green technology innovation on carbon emission performance under different levels of capital misallocation. We find that with the increase in the level of capital misallocation, the regression coefficient of green technology innovation increases, indicating that the higher the degree of capital misallocation is, the greater the negative effect of green technology innovation on carbon emission performance. Then, we use labour misallocation as a threshold variable with which to examine the impact of green technology innovation on carbon emission performance under different levels of labour misallocation. We find that as the degree of labour misallocation increases, the regression coefficient of green technology innovation also increases, indicating that the higher the labour misallocation is, the greater the negative effect of green technology innovation on carbon emission performance. The above empirical results further confirm that both capital misallocation and labour misallocation have inhibitory effects, hindering the effect of the promotion of green technology innovation on carbon emission performance. This finding may be due to the fact that resource misallocation hinders the realization of green technology innovation. On the one hand, green technology innovation requires considerable financial support. However, capital misallocation causes funds to flow into sectors or industries with low productivity, thereby affecting the rational allocation of funds (Kemp-Benedict, 2018). On the other hand, green technology innovation requires a large amount of high-tech talent. However, labour misallocation leads to the loss of high-tech talent, reducing the level of technology innovation, which is not conducive to improving carbon emission performance (Li et al., 2021).

### Robustness test

To verify the validity of the above empirical results, this paper uses DIF-GMM model estimation and replaces the explanatory variables to test the robustness of the results. We use the number of green utility model patents granted to represent green technology innovation. The test results are shown in Tables 7, 8. The robustness results show that green technology innovation helps improve carbon emission performance and that capital misallocation and labour misallocation are not conducive to improving carbon emission performance, findings that are consistent with the previous empirical results.

**Table 7:** The robustness test (1).

Variables	OLS	FE	RE	SYS-GMM
*L.cep*				0.340^***^
				(7.567)
*lngti*	0.254^**^	0.690^***^	0.286^**^	0.896^***^
	(2.155)	(2.614)	(2.060)	(3.795)
*pgdp*	0.191^***^	0.318^**^	0.189^**^	−0.068
	(2.747)	(2.019)	(2.338)	(−0.519)
*eru*	−3.150^***^	−2.665^***^	−2.914^***^	−3.381^***^
	(−4.296)	(−2.801)	(−3.495)	(−4.673)
*open*	−0.553	3.360^***^	−0.375	−1.480
	(−1.614)	(3.266)	(−0.905)	(−1.473)
*gov*	−1.431	−12.860^***^	−2.030	−1.745
	(−1.274)	(−4.697)	(−1.493)	(−1.111)
*fdi*	−0.025	0.059	−0.018	−0.095^*^
	(−0.870)	(1.197)	(−0.505)	(−1.909)
*_cons*	−1.267^*^	−3.537^***^	−1.448^*^	−3.618^***^
	(−1.671)	(−3.635)	(−1.744)	(−2.724)
*AR(1)*				−3.36
				[0.001]
*AR(2)*				1.32
				[0.187]
*Hansen test*				22.44
				[0.972]
*R-squared/Wald test*	0.208	0.117	0.205	635.15^***^
*N*	420	420	420	420

*** *p* < 0.01;

** *p* < 0.05;

* *p* < 0.1.

Figures in () are the *t* values of the coefficients, and figures in [] are the *p* values of the corresponding test statistics. L. is the lag of the variable.

**Table 8:** The robustness test (2).

Variables	DIF-GMM	DIF-GMM	DIF-GMM
*L.cep*	0.115^***^	0.456^***^	0.281^**^
	(4.484)	(4.837)	(2.550)
*lngti*	3.558^***^		
	(8.494)		
*kr*		−9.205^**^	
		(−2.227)	
*lr*			−7.427^**^
			(−1.963)
*pgdp*	−0.617^**^	0.503^***^	0.943^***^
	(−2.239)	(4.544)	(7.711)
*eru*	−5.567^***^	−4.225^***^	−4.817^***^
	(−8.369)	(−8.744)	(−5.879)
*open*	−2.479	1.741	7.626^***^
	(−1.030)	(0.771)	(3.644)
*gov*	−59.937^***^	2.108	−11.770^***^
	(−10.397)	(0.440)	(−5.868)
*fdi*	0.138^***^	0.069^***^	0.149^***^
	(5.814)	(4.534)	(4.602)
*AR(1)*	−3.32	−3.07	−2.99
	[0.001]	[0.002]	[0.003]
*AR(2)*	0.19	1.45	1.15
	[0.846]	[0.148]	[0.251]
*Hansen test*	29.04	23.23	20.91
	[0.852]	[0.332]	[0.464]
*N*	420	420	420

*** *p* < 0.01;

** *p* < 0.05.

Figures in () are the *t* values of the coefficients, and figures in [] are the *p* values of the corresponding test statistics. L. is the lag of the variable.

## Conclusion and policy implications

To improve carbon emission performance, it is very important to enhance green innovation capabilities. Considering that resources are the basis of green technology innovation, resource misallocation influences the effect of the promotion of green technology innovation on carbon emission performance. Based on provincial panel data of China from 2005 to 2018, this study systematically incorporates green technology innovation, resource misallocation and carbon emission performance into the same analytical framework by using panel regression models and a dynamic threshold panel model. The research conclusions are as follows. (1) Green technology innovation plays a significant role in promoting carbon emission performance. However, capital misallocation and labour misallocation are not conducive to improving carbon emission performance. (2) Resource misallocation plays a negative moderating role in the impact of green technology innovation on the improvement of carbon emission performance. The conclusions remain valid regardless of whether capital and labour misallocation are considered individually or together. (3) Under the effect of resource misallocation, green technology innovation has a nonlinear promotion effect on carbon emission performance. Based on the above conclusions, this study puts forward the policy recommendations presented below.

First, the government should continue to strengthen green technology innovation and improve the innovation guarantee system. Moreover, the government should actively lead the green development of emerging industries, advocate the use of green and low-carbon energy in various departments, and accelerate the development of green products. Energy-saving and environmental protection industries and cleaner production industries should be developed, and traditional manufacturing industries should be encouraged to accelerate their green transformation and upgrading.

Second, the market-oriented reform of factors should be deepened, and the optimal allocation of factor resources should be realized. The government should relax its excessive regulation of resources, reduce excessive intervention in the factor market, and release more market vitality. In addition, the rational allocation of factor resources in different departments and regions should be properly guided. Furthermore, it is necessary to improve the green industry financial system, expand multiple financing channels, adjust the profit and loss of funds through the financial system, guide the flow of funds through the price of funds, and improve capital allocation efficiency.

Third, it is very meaningful to solve labour misallocation by strengthening human capital investment; actively cultivating professional and high-tech talent; improving household registration, land systems and social security systems; removing barriers to labour mobility across departments; and reducing labour mobility costs.

Finally, the government should strengthen its antimonopoly policies, investigate and punish acts of unfair competition in accordance with the law, eliminate local protection and market segmentation, accelerate the establishment of a unified national market system and rules, improve resource allocation efficiency, and promote high-quality economic development.

Although this study analyses the tripartite relationship among green technology innovation, resource misallocation, and carbon emission performance, some limitations exist. Future research can be further expanded in the following aspects. First, based on data availability, more detailed city-level data can be used for analysis. Second, the impact of land misallocation and data misallocation on environmental and carbon performance can be further analysed. Third, green technology innovation, resource misallocation, and environmental indicators can be measured through the use of other methods.

## Data availability statement

Publicly available datasets were analyzed in this study. This data can be found at: https://data.stats.gov.cn/.

## Author contributions

MD: writing—original draft, software, methodology, and project administration. QZ: conceptualization, funding acquisition, supervision, and writing—review and editing. YZ: conceptualization, writing—review and editing, supervision, and validation. FL: data curation, formal analysis, writing—original draft, and visualization. All authors contributed to the article and approved the submitted version.

## Funding

This work was financially supported by the Major Program of National Social Science Foundation of China (21&ZD151).

## Conflict of interest

The authors declare that they have no known competing financial interests or personal relationships that could have appeared to influence the work reported in this paper.

## Publisher’s note

All claims expressed in this article are solely those of the authors and do not necessarily represent those of their affiliated organizations, or those of the publisher, the editors and the reviewers. Any product that may be evaluated in this article, or claim that may be made by its manufacturer, is not guaranteed or endorsed by the publisher.
